# To determine the association between asthma severity and hospital admission measured by Pediatric Respiratory Assessment Measure (PRAM) score at Indus Hospital and Health Network, Karachi, Pakistan, 2020-2021

**DOI:** 10.12669/pjms.38.ICON-2022.5783

**Published:** 2022-01

**Authors:** Unaisa Kazi, Saira Gul Rukh, Suha Zawawi, Saba Laila, Mohammad Fareeduddin, Syed Ghazanfar Saleem

**Affiliations:** 1 Dr. Unaisa Kazi, MCPS, Specialist Pediatrics Emergency, Indus Hospital and Health Network (IHHN), Korangi Crossing, Karachi, Pakistan; 2 Dr. Saira Gul Rukh, MBBS, Medical Officer, Emergency Department, Indus Hospital and Health Network (IHHN), Korangi Crossing, Karachi, Pakistan; 3 Dr. Suha Zawawi, MBBS, Emergency Department, Indus Hospital and Health Network (IHHN), Korangi Crossing, Karachi, Pakistan; 4 Dr. Saba Laila Aslam, FCPS, Head of Pediatric Emergency Department, Indus Hospital and Health Network (IHHN), Korangi Crossing, Karachi, Pakistan; 5 Dr. Mohammad Fareeduddin, FCPS, Chair, Pediatric Medicine & Allied, Indus Hospital and Health Network (IHHN), Korangi Crossing, Karachi, Pakistan; 6 Dr. Syed Ghazanfar Saleem, FCPS, Chair Emergency Medicine, Indus Hospital and Health Network (IHHN), Korangi Crossing, Karachi, Pakistan

**Keywords:** Asthma, Paediatric Respiratory Assessment Measure

## Abstract

**Objectives:**

To determine the association between asthma severity and the likelihood of hospitalization by using Pediatric Respiratory Assessment Measure (PRAM) score for pediatric patients who present to the emergency department (ED) with mild, moderate or severe asthma exacerbations and those who received standard intensive asthma therapy.

**Methods:**

This was a retrospective study conducted in children aged between 2 to 14 years. The data was entered and analysed using Statistical Package for the Social Sciences (SPSS) version 21. To be included in the study, the children must have received “intensive asthma therapy” defined as administration of systemic corticosteroids with three albuterol treatments and ipratropium.

**Results:**

A total of 437 patients were enrolled in the study out of which 250 were male and 187 were female. The mean age was 6.1 ± 3.4 years with a minimum age of two and a maximum age of 14 years. The 4-hour PRAM score (AUC = 0.88) overall significantly improved the predictive value of admission (p value <0.001) as compared to the PRAM score calculated at triage (AUC = 0.81).

**Conclusion:**

The 4-hour PRAM score is the best predictor for the need of hospitalization. It is suggested that these results are applied clinically in the pediatric ED to improve patient flow and to better facilitate intensive therapy of patients at triage to decrease the need for hospitalization.

## INTRODUCTION

Asthma is a chronic respiratory disease that affects people of all ages and is characterized by episodic and reversible attacks of wheezing, chest tightness, shortness of breath, and coughing.^1^ ^1^ Estimations show that approximately 30% of asthma-related emergency visits in the pediatric age group result in hospitalization.^2^ In Pakistan, the prevalence of asthma among children ranges from 15 to 20% in different areas of the country.^3^,^4^

Many authentic scoring systems such as, the Paediatric Respiratory Assessment Measure (PRAM) score, Respiratory Rate-Accessory Muscle Use-Decrease breath sounds (RAD) score and Paediatric Asthma Severity score are in use for classifying the severity of asthma, and guiding treatment.^5^,^6^ For PRAM scoring, mechanisms of wheezing, entry of air, scalene muscle contraction, suprasternal retraction and oxygen saturation are all incorporated into a score that is used for children aged between 2 to 17 years that present with acute exacerbation of asthma. The PRAM scoring system has shown to be a quick assessment and differentiating tool with high reliability.^7^ Although authorised scorings that guide evidence-based management are present, there still remains substantial underuse of the proven treatments of asthma.^8^ Besides the emotional burden on patients and families, paediatric asthma also carries a huge financial burden; up to 45% of asthma health care expenses are related to visits to the emergency department (ED) and inpatient hospital care.^9^ Research shows that up to 30% of children who present with severe asthma in the ED are ultimately hospital admissible.^10^

According to research, the use of ’intensive asthma therapy’ comprising systemic corticosteroids with three albulterol treatments and ipratropium 1 hour after triage reduces the duration of ED stay and hospital admission.^11^ Literature shows that standardizing care for asthma patients during early course in the ED helps in optimizing patient care. 9 Early identification of the asthma severity using the PRAM score has the ability to enhance the ED patient flow. Previous literature shows us that there have been efforts to determine the role of PRAM at different hours but there still happens to be a gap in knowledge as most studies do not include PRAM scoring in accordance with administration of standardized evidence-based asthma treatment with complete adherence. Data shows that patients presenting to the ED with mild asthma exacerbations were at low risk of admission.^12^ It has been proven that after initiation of evidence-based treatment, the PRAM scoring is preferable to estimate the likelihood of hospitalization compared with intensive management in the ED. This ability would assist with better management of patient flow in the ED and may also encourage the physicians to administer more aggressive patients earlier in the high-risk population presenting with higher PRAM scores. The main goal of our study was to determine when the PRAM score best predicts the need for patient hospitalization.

## METHODS

A retrospective cohort study was conducted including male and female children between two to 14 years of age that presented to the Emergency Department at The Indus Hospital and Health Network (IHHN) with mild, moderate or severe asthma between October 2018 and March 2019. Two hundred and twenty-seven children were selected using non-probability convenient sampling with a 95% confidence interval and significance level of p<0.05. The data was entered and analysed using Statistical Package for the Social Sciences (SPSS) version 21. Cleaning and coding of data was done prior to analysis. Mean ± standard deviation (STD) was computed for normally distributed continuous variables, while for skewed data, median with interquartile range was observed along with mean ± STD. Normality of data was checked by Shapiro Wilk’s test, histogram and quantile-quantile (Q-Q) plot. On the other hand, frequency with percentage was calculated for categorical variables. To assess the predictive ability of PRAM score for admission, Receiver operator characteristic (ROC) curve were constructed and area under the curve (AUC) was obtained along with best cut-off values for sensitivity and specificity of the PRAM score. This study was approved by IRB and the number is IRD_IRB_2019_09_004

### Sample Selection:

### Inclusion Criteria:

- Children of both genders between two and 14 years of age
- Children presenting with acute asthma exacerbations (defined as triage PRAM score ≥ 4, but < 11)
- Patients with a prior diagnosis of asthma or those who have had three or more episodes of wheezing responsive to beta-2 agonists

### Exclusion Criteria:

- Children with PRAM scores < 4 (mild exacerbation) or > 11
- Hypersensitivity to dexamethasone or oral corticosteroids
- Chronic respiratory conditions such as broncho-pulmonary dysplasia or cystic fibrosis, cardiac, metabolic, or immunologic disease
- History of adrenal suppression
- Patients with a coexisting acute illness such as pneumonia, pertussis, or croup
- Use of oral corticosteroid in the past 14 days
- Exposure to varicella in the previous three weeks in a susceptible child

Using the IHHN Health Management Information System (HMIS), patients were stratified into mild, moderate and severe groups based on the PRAM score on arrival and after four hours of presentation.

### Data Collection:

The data collected during the study includes 1) the PRAM score assessed by physicians on duty at approximately 0, one and four hours or until admission or discharge (depending on the nature of ED care), 2) time of administration of oral steroid; 3) time of inhaled beta-2 agonist, inhaled anticholinergic, and other medications; 4) time of discharge from ED; 5) time taken for the physician to admit the patient; 6) time of admission to inpatient unit; and 8) duration of inpatient stay.

### Data Analysis:

Data was analysed using SPSS version 2.0. and the results were presented as frequency and percentages for qualitative variables and means ± SD for quantitative variables. Chi square test was used to assess the association and a p-value of ≤ 0.05 was considered statistically significant.

## RESULTS

A total of 437 patients were enrolled; 250 (57.2%) were male, while 187 (42.8%) were female. The mean age was 6.1 ± 3.4 years with a minimum age of two and a maximum age of 14 years. Overall, 288 (65.9%) of the children had inspiratory and expiratory wheeze on auscultation. Air entry was normal in a total of 334 (76.4%) patients. Table-I.

**Table I:** Baseline demographic and clinical parameters of asthmatic children n=437.

		n (%) / Mean STH & Median, IQR
Gender	Male	250(57.2)
	Female	187(42.8)
Age in years		6.1 ± 3.4 & 5, 5.5
Length of stay		8.3 ± 9.3 & 4, 12
Pram score		4.6 ± 2.5 & 5, 5
Wheeze	Inspiratory and expiratory	288(65.9)
	Expiratory	124(28.4)
	Absent	21(4.8)
	Audible without Stethoscope	4(0.9)
Air entry	Normal	334(76.4)
	Decreased at basis	73(16.7)
	Widespread decreased	30(6.9)
Oxygen saturation	> 95%	247(56.5)
	92% - 95%	122(27.9)
	< 92%	68(15.6)

On calculating the PRAM score at arrival, we observed that 213 (48.7%) of the children had mild asthma, while moderate and severe asthma was present in 208 (47.6%) and 16 (3.7%) patients, respectively. Majority of the children needed nebulization 420 (96.1%) and steroids 294 (67.3%). On reassessment of the patients after one hour in the ED, an overall improvement in the severity was observed with just 2 (0.5%) of 16 patients with severe asthma at one hour Similarly, 114 out of 208 children who had initially presented with moderate asthma improved after being treated in the ED. On assessing the PRAM score at four hours, we observed that out of 94 children with moderate asthma, only 59 (13.9%) children were left with moderate disease while 303 (69.3%) were discharged after treatment within four hours. 62 (14.2%) patients were referred out to other health facilities, 65 (14.9%) were admitted in our hospital out of which 53 (81.5%) were admitted in the paeds high dependency unit. Table-II.

**Table II:** Assessment and management of patients with help of PRAM score n (%).

PRAM score at arrival of patients	Mild (1-4)	213(48.7)
	Moderate (5-8)	208(47.6)
	Severe (9-15)	16(3.7)
PRAM score at 1st hour	Mild (1-4)	341(78)
	Moderate (5-8)	94(21.5)
	Severe (9-15)	2(0.5)
PRAM score at 4 hour	Mild (1-4)	171(39.1)
	Moderate (5-8)	59(13.5)
Nebulization		420(96.1)
Use of Steroids		294(67.3)
Use of MgSO4		137(31.4)
Triage	P1	13(3)
	P2	337(77.1)
	P3	82(18.8)
	P4	5(1.1)
Plan for patient	Admission	65(14.9)
	Discharge	303(69.3)
	LAMA	7(1.6)
	Refer out	62(14.2)
Admitting place	General ward	4(6.2)
	PHDU	53(81.5)
	PICU	5(7.7)

**Fig.1:**
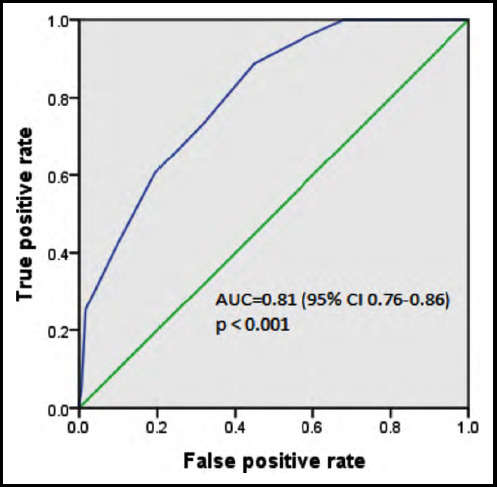
ROS Curve.

The ROC curve for PRAM on arrival to the ED i.e., 0 time showed an AUC of 0.81 (95% CI 0.76 – 0.86), depicting that the PRAM scoring system has good capability in predicting the admission probability of asthmatic patients. At 0 hour, at the cut-off of 5.5, the PRAM score showed a sensitivity of 73.2% to predict true admission and a specificity of 68.3% to detect true cases with no need of admission. These findings are highlighted in Fig.1 and Table-III(a).

**Table III (a):** Sensitivity and specificity of PRAM score at arrival.

Positive if Greater Than or Equal To^a^	Sensitivity	Specificity
-1.0	1.0	1.0
0.5	100.0	3.6
1.5	100.0	16.9
2.5	100.0	32.0
3.5	95.8	41.5
4.5	88.7	55.2
5.5	73.2	68.3
6.5	60.6	80.6
7.5	42.3	90.2
8.5	25.4	98.4
9.5	4.2	99.5
10.5	1.4	100.0
12.0	0.0	100.0

**Fig.2:**
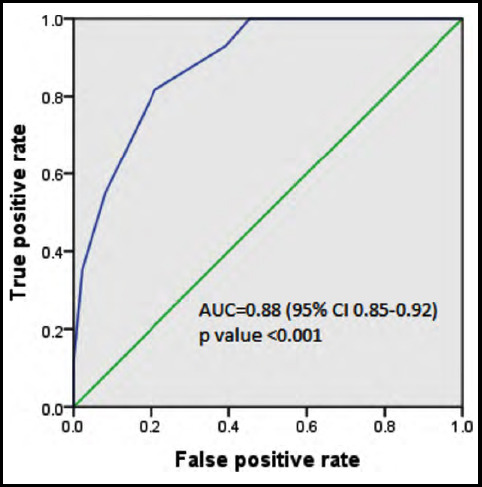
ROS Curve.

When we recalculated the PRAM score at one hour, we observed that there was an improvement in the predicted capacity of PRAM score as AUC was increased up to 0.88 (95% CI 0.85-0.92) with the statistically significant p value of <0.001. In the same way sensitivity and specificity were also increased from 73.2% to 81.7% and 68.3% to 79% respectively at the cut-off level of 4.5. Fig.2 and Table-IIIb.

**Table III (b):** Sensitivity and specificity of PRAM score at one hour.

Positive if Greater Than or Equal To^a^	Sensitivity	Specificity
-1.0	1.0	1.0
0.5	100.0	37.3
1.5	100.0	51.3
2.5	100.0	65.6
3.5	93.0	60.8
4.5	81.7	79.0
5.5	54.9	91.9
6.5	35.2	97.8
7.5	11.3	100.0
8.5	4.2	100.0
9.5	1.4	100.0
11.0	0.0	100.0

## DISCUSSION

Asthma is a heterogenic condition that is underdiagnosed and undertreated despite that the skills needed to diagnose it are readily attainable and effective treatments are available.^13^ Chronic lower airway inflammation is known to be more common in individuals that also have inflammatory disorders of the upper airway.^13^ A retrospective observational cohort study was carried out in the pediatric emergency department of the Indus Hospital and Health Network (IHHN) on PRAM scoring and its predictive capacity at different hours for the need of hospitalization.

The patients were received at triage for assessment of vitals followed by transfer to the pediatric ED for assessment. PRAM scoring was done at the first encounter of the patients with physicians in the ED after triage and was labelled as “0 hour” followed by administration of standard evidence-based asthma treatment. To our knowledge, there is one such study that includes patients receiving standardized asthma therapy.^14^ The null hypothesis of the study was defined as no association between asthma severity and the possibility of hospital admission for pediatric patients through the ER which was rejected as a statistically significant difference was observed. In a low-resource setting, upon reassessing the patients at the 1^st^ hour after administering treatment, a remarkable increase was reported in the predictive capacity of PRAM scoring with increased sensitivity and specificity in comparison to the PRAM score calculated at “0 hour”.

According to literature, besides PRAM, a number of clinical scores have been studied such as, the Pediatric Asthma Severity Score (PASS), the Clinical Asthma Score (CAS), the Asthma Severity Scare (ASS) and the Pulmonary score with the Pulmonary Index (PI). The Pulmonary score with PI is the score most widely used in asthma clinical trials.^15^ A study conducted in the ED of the Aga Khan University Hospital, Karachi investigated the outcomes of children aged between one month to 16 years using the Clinical Respiratory Score (CRS) and concluded that patients with higher scores were more likely to be admitted to the pediatric critical care unit.^16^ As compared to the PRAM score, CRS takes into account the mental status and appearance of the child and does not require expert training to use.

Another prospective study studied the comparison between Wood’s and PRAM score to determine which was a better predictor of severity of childhood asthma exacerbations and the results showed that both scores were promising in predicting the outcome and severity in children.^17^

It is therefore suggested that the PRAM score should be used in the assessment of asthma severity in the pediatric population and should be recalculated at hour one after administering treatment. By using this assessment tool, physicians may be able to predict hospitalizations better and admit sicker patients with higher scores earlier, freeing beds in the ED and assisting in improving patient flow. Implications of this study’s findings have the potential to improve the emergency department’s throughput, reliability and quality of patient care for children with asthma.

### Strengths and Limitations:

This was a retrospective study that collected data from the Health Management Information System (HMIS) and the extraction of such data is dependent on the level and accuracy of documentation in the medical record. Due to patient improvement by the 1^st^ hour, it was possible to determine the disposition of the patient which led to a lot of the participants being discharged before the mark of the 4^th^ hour. Moreover, the study only included children from one center in Karachi which is a limited cohort of the general pediatric population of Pakistan. The scoring and decision of disposition of the patient was dependent upon the clinical judgment of the treating physician. Lastly, Children of ages < 2 and > 14 years were not included in the study and the PRAM scores for all hours were not available. However, the study consisted of a large sample size with an almost equal distribution of genders and a variety of age groups were still included.

## CONCLUSION

It is concluded that the use of PRAM at hour-1, measured after the initiation of evidence-based therapy, is the best predictor of hospitalization. It should therefore be adopted in routine use of pediatric asthmatic patients. The PRAM scoring system has shown credibility in improving the Emergency Department patient flow and managing patients who have not received maximum intensive therapy in order to initiate more aggressive methods and prevent hospitalization.

### Author contribution:

**UK:** Substantial contribution towards the concept and design of the study, initial write up, revised it critically for important intellectual content.

**SGR:** Data collection, literature search, contributed the initial write-up.

**SZ:** Substantially contributed towards data cleaning, analysis, interpretation and result section write up.

**SL, MF:** Literature search and discussion write up.

**GS:** Substantial contribution towards the concept and design of the study, final editing, revised it critically for important intellectual content.
